# *Acanthospermum australe* Extract Inhibits the Chaperone Activity of *Plasmodium falciparum* Heat Shock Protein 70-1

**DOI:** 10.3390/microorganisms13092195

**Published:** 2025-09-19

**Authors:** Ntombikhona Appear Koza, Ntokozo Nkosinathi Myeza, Heinrich Hoppe, Rebamang Anthony Mosa, Abidemi Paul Kappo, Mthokozisi Blessing Cedric Simelane, Andrew Rowland Opoku

**Affiliations:** 1 Department of Biochemistry and Microbiology, University of Zululand, KwaDlangezwa 3886, South Africa; ntokozomyz@gmail.com (N.N.M.); opokua@unizulu.ac.za (A.R.O.); 2 Department of Biochemistry and Microbiology, Rhodes University, P.O. Box 94, Grahamstown 6140, South Africa; h.hoppe@ru.ac.za; 3 Department of Biochemistry, Genetics and Microbiology, University of Pretoria, Hatfield 0028, South Africa; rebamang.mosa@up.ac.za; 4 Department of Biochemistry, University of Johannesburg, P.O. Box 524, Auckland Park 2006, South Africa; akappo@uj.ac.za (A.P.K.); msimelane@uj.ac.za (M.B.C.S.)

**Keywords:** *Plasmodium*, medicinal plants, heat shock proteins

## Abstract

The resistance of malaria parasites towards the current antimalarial therapies continues to fuel the search for new antimalarial drugs, preferably from natural sources. This study aimed to investigate the potential of the dichloromethane extract of *Acanthospermum australe* to inhibit *Plasmodium falciparum* heat shock protein 70-1 (*Pf*Hsp70-1). The plasmodium lactate dehydrogenase (pLDH) assay was used to determine the antiplasmodial activity of the crude extract against the chloroquine-sensitive *P. falciparum* strain 3D7. The inhibitory effect of the plant extract on the chaperone activity of *P. falciparum* heat shock protein 70-1 (*Pf*Hsp70-1) was determined using the ATPase, thermally induced luciferase and malate dehydrogenase (MDH) assays. The extract showed a significantly high activity against *P. falciparum* strain 3D7 with an IC_50_ value of 1.3 µg/mL. A decrease in thermally induced aggregation of MDH and luciferase was observed when each of the proteins was incubated with *Pf*Hsp70-1 only. However, an increased protein aggregation was observed when the proteins were incubated with *Pf*Hsp70-1 in the presence of the plant extract. The extract also exhibited inhibitory activity on the ATPase activity of *Pf*Hsp70-1. The results obtained from this study suggest that *A. australe* extract contains compounds that could target malaria parasite Hsp70 functions.

## 1. Introduction

Malaria continues to be among the major human health concerns worldwide, with many developing countries being the most affected [1]. Among the six plasmodium species that cause malaria, *Plasmodium falciparum* is the deadliest and responsible for killing about 200,000 children every year, and 7 in 10 deaths are among children under 5 years old [2,3]. The effectiveness of the current antimalarial drugs has declined due to their short half-life, low bioavailability, and the emergence of the resistant *P. falciparum* strain [4,5]. As long as malaria infection and the spread of drug resistance continue, the search for antimalarial drugs with improved efficacy and/or novel mechanisms of action is ongoing. While the current antimalarial drugs, such as chloroquine and artemisinin derivatives, act by inhibiting hemozoin formation and generation of free radicals [6,7], there are no reports on the effects of these drugs on the parasite heat shock proteins. These proteins function as molecular chaperones crucial to the parasite’s survival.

Heat shock protein 70 (Hsp70), one of the highly conserved molecular chaperones, is the most studied homologue. Generally, Hsp70 is ubiquitously expressed in cells in response to normal and stressful conditions. *P. falciparum* consists of different Hsp70 (*Pf*Hsp70-1, *Pf*Hsp70-2, *Pf*Hsp70-3, *Pf*Hsp70-y, *Pf*Hsp70-z, and *Pf*Hsp70-x) proteins that have been well-characterised as molecular chaperones with ATPase activity [8]. These *Pf*Hsp70 proteins have been reported to maintain growth and development during the parasite’s life cycle. *Pf*Hsp70-1, one of the cytosolic *Pf*Hsp70 homologues, provides cyto-protection to the parasite during stressful conditions and the pathogenicity of the malaria parasite [9,10]. *Pf*Hsp70-1 responds to heat shock during the transition of the malaria parasite from the cold vector (mosquito) to increased human body temperature [11]. The discovery of new antimalarials that inhibit *Pf*Hsp70-1 functions could be crucial for malaria treatment. Therefore, there is an increasing interest in searching for novel antimalarial drugs from natural sources such as medicinal plants.

*Acanthospermum australe* (*Asteraceae*) is traditionally used to treat parasitic worms, skin diseases, and diarrhoea [12]. The plant is also used by some traditional healers in the KwaZulu-Natal province of South Africa to treat fever and malaria. Nethengwe et al. (2012) reported antipyrexia and preliminary antiplasmodial properties of the dichloromethane extract of the plant [13]. The current study aimed to confirm the antiplasmodial activity of the plant extract and further evaluate its inhibitory effect on *Pf*Hsps70-1 activities.

## 2. Materials and Methods

### 2.1. Chemicals and Reagents

Sodium carbonate and dimethyl sulfoxide (DMSO) were purchased from Merck (Darmstadt, Germany). Chloroquine, kanamycin, bis-acrylamide, sodium dodecyl sulfate (SDS), isopropyl-β-D-thiogalactopyranoside (IPTG), Coomassie blue R 250, phenylmethylsulphonyl fluoride (PMSF), lysozyme, dithiothreitol (DTT), ammonium molybdate, 2-mercaptoethanol, tris (hydroxymethyl)aminomethane, and polyvinylidene fluoride (PVDF) were all purchased from Inqaba Biotechnical Industries (Pretoria, South Africa).

### 2.2. Plant Collection and Extract Preparation

Fresh plant material of *Acanthospermum australe* (Loefling) Kuntze was collected at Esikhawini (Ndindima) (28.88261, 31.94971), KwaZulu-Natal, South Africa. Prof. N.R. Ntuli, from the Botany Department of the University of Zululand, confirmed the plant’s identity. The air-dried plant material (leaves and stems) was ground into powder (2 mm mesh). The powdered plant material (269 g) was first defatted with *n*-hexane and extracted with dichloromethane (1:5 *w*/*v*) (24 h on a platform shaker at room temperature). The filtrate was concentrated under reduced pressure at 40 ± 2 °C using a rotary evaporator (Heidolph Instruments, Schwabach, Germany) to obtain crude dichloromethane (DCM) extract (15 g). The crude extract was kept in the fridge (4 °C) until required.

### 2.3. Gas Chromatography–Mass Spectrometry Analysis

The phytochemical composition of the plant extract was analysed using gas chromatography (GC). The GC was connected to a mass spectrometer furnished with Elite-1 and a fused silica capillary column. The compounds were detected by employing an electron ionisation system with an ionisation energy of 70 eV. Helium gas was used as a carrier at a constant flow rate of 1 mL and a 2 μL injection volume. The injector was maintained at a temperature of 250 °C and ion-source temperature of 280 °C. The oven temperature was programmed to hold for 2 min at 110 °C, further increased by 10 °C/min interval until it reached 200 °C; then it was increased by 5 °C/min to 280 °C, and the isothermal ended after 9 min at 280 °C. The mass spectra (MS) were obtained at 70 eV, with a scanning interval of 0.5 s and fragments from 45 to 450 Da. The overall running time of the GC-MS (Perkin Elmer, Shelton, CT, USA) was 30 min for each sample. Turbomass 5.1 software for the mass spectra mass spectra and chromatograms were used to obtain the compound data. These compounds were matched with database compounds of the National Institute of Standards and Technology library (Gaithersburg, MD, USA) (NIST08.LIB).

### 2.4. Plasmodium Lactate Dehydrogenase (pLDH) Assay

The plasmodium lactate dehydrogenase (pLDH) assay described by Makler et al. (1993) was adapted to determine the in vitro antiplasmodial activity of the extract on the chloroquine-sensitive strain of *Plasmodium falciparum* (3D7) [14]. The *P. falciparum* 3D7 cells were maintained in RPMI 1640 medium containing glutamine and HEPES. The medium was further supplied with albumax, glucose, hypoxanthine, gentamycin, and hematocrit human red blood cells. The parasites were cultured at 37 °C under an atmosphere of 5% CO_2_, 5% O_2_, 90% N_2_, in sealed T25 culture flasks. Different concentrations (10 and 50 µg/mL) of the plant extract were prepared in DMSO (<1%). Each of the different concentrations of the extract was added to the parasite cultures in the respective wells of the 96-well plate and incubated for 48 h with a mixture of Malstat and NBT/PES solutions in the 96-well plate. The absorbance of the purple-coloured product was measured at 580 nm.

### 2.5. Transformation and Expression of Recombinant PfHsp70-1 Protein

Competent *E. coli XL1 blue* cells (Qiagen, Hilden, Germany) were transformed with the pQE30/*Pf*Hsp70-1 plasmid. Briefly, 1 μL of the plasmid DNA was mixed with 100 μL of pre-thawed competent bacterial cells and incubated on ice for 30 min and then heat-shocked at 42 °C for 45 s to allow for the uptake of the plasmid DNA by the bacterial cells. Immediately after this, further incubation of the mixture was done on ice for 5 min, followed by the addition of 900 μL of pre-warmed LB broth without ampicillin. This mixture was then further incubated at 37 °C for 60 min. Thereafter, 100 μL of the transformed cells were then spread out on LB–agar plates supplemented with 100 μg/mL ampicillin and further incubated at 37 °C for 16 h. The appearance of colonies on the LB–agar plates after this period confirmed the successful transformation of the cells. Following this, a single colony from the LB–agar plates was then used to inoculate 100 mL of LB broth supplemented with 100 μg/mL ampicillin to begin the recombinant expression of the *Pf*Hsp70-1 protein using previously described protocols [15,16]. The protein expression was induced with 1 mM isopropyl-β-D-thiogalactopyranoside (IPTG), and 1 mL of the culture was collected with Eppendorf vials from 1 to 6 h. All the collected cultures in Eppendorf vials were harvested by centrifugation at 5000× *g* for 30 min at 4 °C, and the cell pellets were resuspended in sodium dodecyl sulfate polyacrylamide gel electrophoresis (SDS-PAGE) sample buffer and electrophoresed on a 12% SDS polyacrylamide gel. The destained gel was visualised with the Chemi-Doc Imaging system (Bio-Rad, Hercules, CA, USA). The expression of pQE30/*Pf*Hsp70-1 was confirmed with Western blot using the Trans-blot Turbo system.

### 2.6. Purification of Expressed PfHsp70-1 Protein

The purification of recombinant *Pf*Hsp70-1 containing a His-tag was prepared under native purification protocol [17]. Briefly, Poly-(ethyleneimine) (1%) was added to the harvested pellets (*E. coli* BL21 cells) containing the expressed *Pf*Hsp70-1 protein for nucleic acid precipitation. Thereafter, the cell pellets were resuspended in freshly prepared lysis buffer. The resultant suspension was mixed thoroughly and followed by sonification to break open the cells. The cleared total bacteria were collected by centrifugation at 12,000× *g* for 30 min at 4 °C. The clear supernatant containing His-fusion *Pf*Hsp70-1 protein was loaded onto a 10 mL Nickel-charged nitrilotriacetic acid resin (Ni-NTA) and gently mixed with Ni-NTA slurry on a shaking platform for 4 h at 4 °C. The flow-through was collected in 10 mL Eppendorf tubes, and the resins were washed out with wash buffer. This was followed by eluting the bound protein using native elution. The eluted His-tagged *Pf*Hsp70-1 protein fractions were analysed with a 12% SDS-PAGE gel and Western blot analysis. The pure bands from the gel confirmed the purity of the *Pf*Hsp70-1 protein. Pure eluted protein was desalted, and the eluents were added to the Snake-Skin tubing and pre-soaked in a dialysis buffer. The generated purified *Pf*Hsp70-1 protein concentration was determined by the NanoDrop ND 2000 spectrophotometer (Thermo Fisher Scientific, Waltham, MA, USA).

### 2.7. Malate Dehydrogenase and Luciferase Aggregation Assay

The inhibitory activity of the plant extract on the chaperone function of *Pf*Hsp70-1 was investigated using thermally induced aggregation assays of malate dehydrogenase (MDH) and luciferase [18]. The thermal-induced malate dehydrogenase MDH aggregation assay was initiated by allowing the HEPES buffer (20 mM HEPES-KOH, pH 7.5, 50 mM NaCl) to equilibrate for 10 min at 45 °C. After 10 min, the *PfHsp70*-1 (10 µM) and varying concentrations (0–25 µg/mL) of the plant extract were added to the buffer. For the assay controls, 2 µg/mL of bovine serum albumin (BSA) and 10 mM of polymyxin B (PMB) were used as negative and positive controls, respectively. The aggregation-prone model protein MDH (2 µM) was added last to the assay mixture. The aggregation reaction for MDH was incubated at 45 °C for 45 min. The thermal-induced protein aggregation was monitored by reading absorbance at 360 nm using the Synergy HT microplate reader.

The thermal-induced luciferase aggregation assay was conducted as described for the MDH assay with slight modifications. The HEPES buffer (20 mM HEPES-KOH, pH 7.5, 5 mM NaOA_C_, 50 mM KCl, 5 mM MgCl_2_, and 5 mM β-mercaptoethanol) was used, and the reaction mixture was incubated at 45 °C for 30 min. The thermally induced protein aggregation was monitored by reading absorbance at 340 nm.

### 2.8. ATPase Assay

The ATPase assay was used to determine the inhibitory activity of the plant extract on the ATPase activity of *Pf*Hsp70-1 [19]. In this assay, the reaction mixture contained HDKM buffer (10 mM HEPES-KOH, pH 7.5, 0.5 mM DTT, 2 mM MgCl_2_, and 100 mM KCl), 1 mM PfHsp70-1, and the extract at varying concentrations (0–25 µg/mL). PMB (1–4 mM) was used as the positive control. The mixture was preincubated at 37 °C for 5 min, and the reaction was initiated by adding 5 mM of ATP. The reaction was allowed to occur at 37 °C for 4 h with collections of 50 μL aliquots at 1 h intervals. To every 50 μL aliquot collected, an equal volume of 10% SDS was added to terminate the reaction. This was followed by an addition of 50 μL of each of 9% ascorbic acid and 1.25% ammonium molybdate. The absorbance of the released inorganic phosphate (P_i_) was read at 660 nm using the UV-1800 spectrophotometer (Shimadzu, Kyoto, Japan). The concentration of the released P_i_ was determined from the calibration curve of potassium phosphate (KH_2_PO_4_) and was expressed as nmol/min/mg.

### 2.9. UV-Vis Spectrometer Analysis

The Ultraviolet-visible (UV-vis) spectrophotometer method described by Opoku et al. (2019) was followed with minor modifications to analyse the conformational changes in *Pf*Hsp 70-1 protein upon its interaction with the plant extract [17]. A purified *Pf*Hsp70-1 protein (1 mM) was exposed to 5 mM and 10 mM of the plant extract at room temperature for 30 min. The spectrum readings of *Pf*Hsp70-1 protein only and protein with extract were determined by UV-vis using 10 scans for every sample at a wavelength region of 200–400 nm. The UV-vis analysis was controlled by 1 mg/mL bovine serum albumin (BSA) as a negative control and 1 mM PMB as a positive control. BSA was used instead of *Pf*Hsp70-1 protein, and PMB instead of the plant extract. The absorption peaks were analysed with OriginPro 8.5 software.

### 2.10. Statistical Analysis

Unless stated otherwise, all experiments were repeated three times. Data generated were entered into Microsoft Excel^®^ or Origin 8 and analysed by calculating the means and standard deviation. The results are reported as mean ± SD. Multiple comparisons of the data were performed by one-way analysis of variance (ANOVA) followed by Dunnett’s *post hoc* test using Graph Pad Prism version 6.01. The values were considered statistically significant where *p* ≤ 0.05.

## 3. Results

### 3.1. Chemical Profiling of the DCM Extract Using GC-MS

The GC-MS analysis of the DCM extract recorded a total of 185 compounds. About 11 were considered major (peak area ≥ 0.2%) compounds in the extract. These include 5-Benzofuranacetic acid, 6-ethenyl-2,4,5,6,7,7a-hexahydro-3,6-dimethyl-à-methylene-2-oxo-, methyl ester, Methyl 10,12-pentacosadiynoate, Fluoxymesterone, and Estra-1,3,5(10)-trien-17á-ol (Table 1).

### 3.2. Antiplasmodial Activity

The antiplasmodial activity of the plant extract was determined against *P. falciparum* strain 3D7, and the results are shown in Table 2. The extract, at only 10 µg/mL, exhibited significantly high antiplasmodial activity (killing 72% of the cells) with an IC_50_ value of 1.3 µg/mL.

### 3.3. The Expression and Purification of PfHs70-1 Recombinant Protein

**Figure 1:**
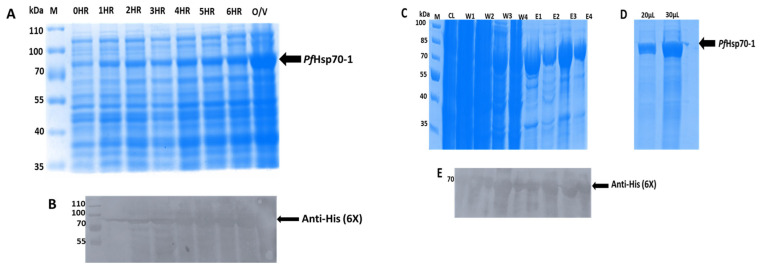
Expression and purification of the *Pf*Hsp70-1 protein. (**A**) Expression of the *Pf*Hsp70-1 protein in *E. coli* BL21 cells: lane M-molecular weight marker (kDa); lane 0 HR-*Pf*Hsp70-1 expression without IPTG; lanes 1-6 HR and O/V-IPTG-induced *Pf*Hsp70-1 expression. (**B**) Western blot of the expressed *Pf*Hsp70-1. (**C**) Purification of the *Pf*Hsp70-1 protein; lane CL, *Pf*Hsp70-1 clear lysate, lane W1-W4 wash samples, and lane E1–E4 were the eluted *Pf*Hsp70-1 protein. (**D**) Re-purification of E1-4. (**E**) Western blot analysis of purified *Pf*Hsp70-1 (Supplementary data available).

SDS-PAGE gel (12%) was used to resolve the recombinant expression of *Pf*Hsp70-1 protein in *E. coli BL21* cells. The gel showed a successful expression profile of the *Pf*Hsp70-1 protein, which indicated a size of ~74 kDa (Figure 1A). The protein bands were observed at approximately 74 kDa in the fractions (0HR to O/V), but the IPTG-induced bacterial cell lysates showed more expressed bands than the uninduced bacterial cell lysates. SDS-PAGE gel showed that the protein was not washed off with the wash buffer, in wash 1 (W3-4). However, elutions 1-4 showed high binding specificity of the *Pf*Hsp70-1 protein to Nickel NTA–agarose beads, but the protein was not 100% pure (Figure 1C). Therefore, the elution from E1-4 was further combined and washed, eluted with elution buffer containing less imidazole, and the expressed *Pf*Hsp70-1 protein appeared to be successfully purified (Figure 1D). Western blot analysis confirmed that the purified *Pf*Hsp70-1 protein migrated at the right molecular weight of 74 kDa (Figure 1E). The concentration of *Pf*Hsp70-1 purified protein was recorded as 0.8 mg/mL.

### 3.4. The Extract Suppresses the Chaperone Activity of PfHsp70-1

**Figure 2:**
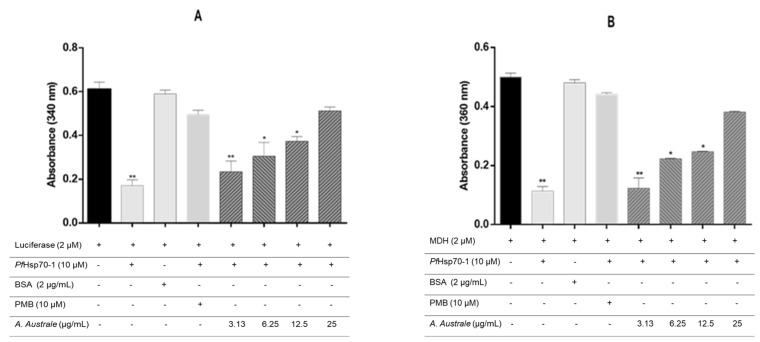
Effect of the extract on the chaperone activity of *Pf*Hsp70-1. The effect of the extracts (3.13, 6.25, 12.5, 25 µg/mL) was tested on the thermally induced aggregation of (**A**) luciferase and (**B**) MDH. Polymyxin B and BSA were used as positive and negative controls, respectively. Data were expressed as mean ± SD (*n* = 3); * *p* < 0.05 and ** *p* < 0.001 compared to model control (luciferase or MDH only). (+) present, (-) absent.

Incubation of the proteins (MDH and luciferase) in the presence of *Pf*Hsp70-1 only resulted in a significant decrease in the thermally induced aggregation of the proteins. However, protein aggregation increased when the model proteins (luciferase and MDH) were incubated with *Pf*Hsp70-1 in the presence of the plant extract (Figure 2A,B). The extract showed a concentration-dependent effect on the thermally induced protein aggregation. The presence of the extract at the highest concentration of 25 µg/mL resulted in the thermally induced luciferase aggregation that was highly comparable to that of PMB (a known inhibitor of *Pf*Hsp70-1) at 10 mM.

### 3.5. A. australe Extract Inhibits PfHsp70-1 ATPase Activity

**Figure 3:**
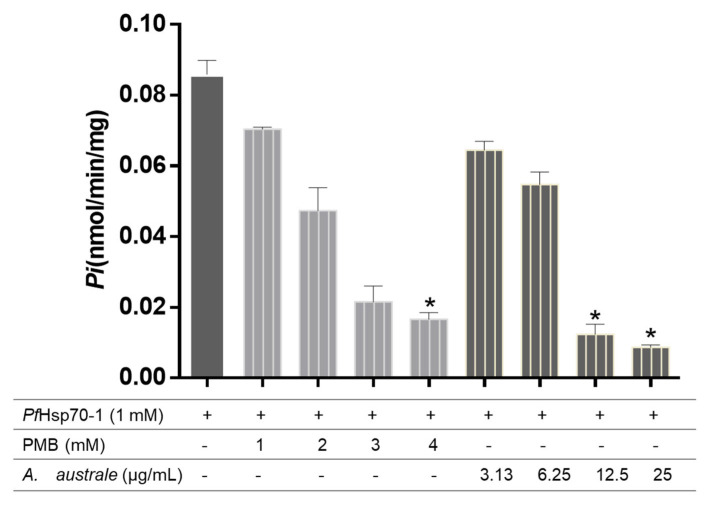
Effect of the extract on the basal PfHsp70-1 ATPase activity. A mixture of *Pf*Hsp70-1, ATP, and the extract was incubated at 37 °C for 4 h. PMB was used as the positive control. Data were expressed as mean ± SD *(n* = 3), * *p* < 0.05 vs. *Pf*Hsp70-1 only. (+) present, (-) absent.

The results of the plant extract on basal ATPase activity are shown in Figure 3. The results showed that the plant extract inhibited the *Pf*Hsp70-1 ATPase activity. While an increase in the concentration of P_i_ released (~0.083 nmol/min/mg) was observed when ATP was incubated with *Pf*Hsp70-1 only, the presence of the plant extract (at 25 µg/mL) significantly decreased the concentration of P_i_ released (~0.009 nmol/min/mg). This was even lower than the amount of P_i_ released (~0.018 nmol/min/mg) when ATP was incubated with *Pf*Hsp70-1 in the presence of PMB at 4 mM.

### 3.6. UV-Vis Analysis of the Interaction of PfHsp70-1 with the Plant Extract

**Figure 4:**
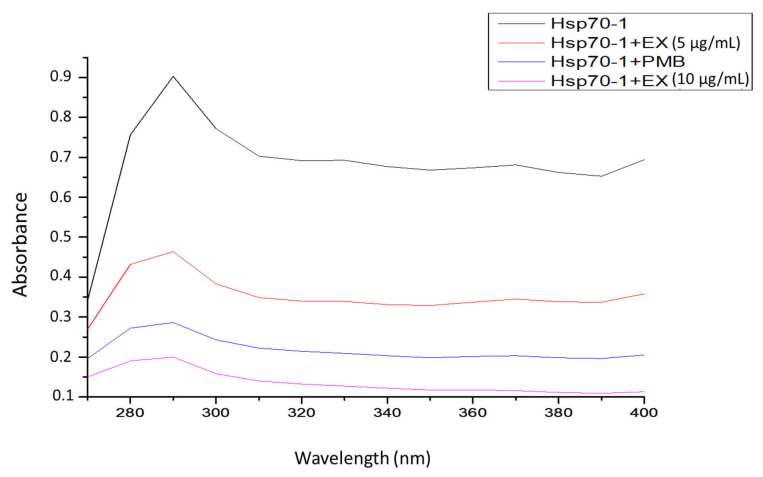
UV-vis absorption spectra of *Pf*Hsp70-1 in the presence of polymyxin B (PMB, 1 mM) and the plant extracts (EX) at 5 µg/mL and 10 µg/mL.

UV–visible spectrum was used to determine the binding effect of the extract on *PfHsp70*-1 protein. The results from UV-vis showed that *Pf*Hsp70-1 protein alone had a high peak with an absorbance of 0.897 nm at a wavelength of 290 nm, showing the exposed aromatic rings. However, when *PfHsp70*-1 protein was exposed to the extract three times, remarkably reduced absorbance (0.203 nm) was observed at 290 nm (Figure 4 and Table 3).

## 4. Discussion

The current study sought to confirm the antiplasmodial activity of the plant extract and further evaluate its effect on the chaperone activity of the *P. falciparum* heat shock protein70-1 (*Pf*Hsps70-1). Since the medicinal properties of plants are attributed to their phytochemical composition, the DCM extract of *A. australe* was first analysed for its phytochemical composition. The GC-MS data revealed chemical diversity, which included compounds such as Methyl 10,12-pentacosadiynoate, 1,6-Heptadien-4-ol, and Octadecanoic acid as major compounds (Table 1). Though the reported antimicrobial activities of some of these compounds [21,24,30] do not necessarily indicate their antiplasmodial activity, the presence of 1,6,10-Dodecatrien-3-ol, 3,7,11-trimethyl-, known for antimalarial activity [26] encouraged investigation of the antiplasmodial potential of the extract.

The antiplasmodial activity of the *A. australe* extract was investigated on *P. falciparum* strain 3D7. According to Waiganjo et al. (2020), high in vitro antiplasmodial activity of crude extract is shown with IC_50_ ≤ 10 µg/mL, moderate activity with IC_50_ value 11–50 µg/mL, and low activity with IC_50_ ≥ 100 µg/mL [37]. The low IC_50_ value (1.3 µg/mL) exhibited by the extract supported the recorded high percentage mortality of the parasite upon exposure to the extract at 50 µg/mL (Table 2), indicating its high potency against the malaria parasite. The obtained results are consistent with those previously reported by Nethengwe et al. [13]. The high activity of the extract could be attributed to its phytochemical composition, which includes 11-Trimethyl-1,6,10-dodecatrien-3-ol (Nerolidol). Nerolidol is known to inhibit the red blood cell stages of the malarial parasite [26,27].

*Pf*Hsps70-1 is very important in the life cycle of the malarial parasite as it provides cyto-protection to the parasites during stressful conditions and the pathogenicity of the malaria parasite [9,10]. The inhibitory activity of the extract on the *PfHsp70-1* chaperone function was determined. The observed failure of *Pf*Hsp70-1 to suppress the thermally induced aggregation of MDH and luciferase in the presence of the plant extract (Figure 2) indicated the inhibitory activity of the extract on the chaperone activity of the *Pf*Hsp70-1 protein. It was postulated that the presence of small inhibitor molecules in the extract might not only inhibit the *Pf*Hsp70-1 chaperone activity but also inhibit its association with other co-chaperone associates. Since the current clinical antimalarial drugs (Quinoline and artemisinin derivatives) are not known to target the parasite heat shock proteins, the inhibitory activity of *A. australe* extract on the *Pf*Hsp70-1 chaperone functions could be crucial in preventing the parasite’s development and survival within the human host. The potential of the extract to also target the asexual blood stage of the parasite, especially the trophozoite formation, could be key in malaria treatment.

The inhibitory effect of the extract was further determined on the basal ATPase activity of *Pf*Hsp70-1. The N-terminal ATPase domain of the *Pf*Hsp70-1 protein is vital for the chaperone functional cycle. This cycle releases the inorganic phosphate (P*i*) during ATP hydrolysis, which allows PfHsp70-1 to keep the client protein folded and prevent protein aggregation [38]. The observed lower P*i* released when ATP was incubated with *Pf*Hsp70-1 in the presence of the plant extract (Figure 3), compared with *Pf*Hsp70-1 only, suggests that the extract inhibited ATPase activity of *Pf*Hsp70-1 protein. Similar findings were also reported from the extracts of *Pterocarpus angolensis* and *Ziziphus mucronate* [10].

UV-vis spectroscopy was used to analyse some conformational changes in the PfHsp70-1 structure upon interaction with the extract. The aromatic amino acids (tryptophan and tyrosine) contribute to strong UV absorption of approximately 240–300 nm wavelength [39]. A decrease in absorption strength when the protein was incubated with the extract (Figure 4 and Table 3) showed that the extract disrupted the *Pf*Hsp70-1 protein stability. This suggests that certain compounds in the extract were able to bind aromatic side chains of the protein’s amino acid residues and thus change its original structure.

The high in vitro antiplasmodial activity of the extract, its inhibitory effect against *Pf*Hsp70 function, and the observed ability to disrupt protein stability indicate that the crude extract contains compounds with good biological activity. The observed effects could be due to the additive and/or synergistic interactions among multiple compounds in the extract. The isolation of these active compounds from the extract and confirming their bioactivities could help identify the key bioactive components responsible for the observed bioactivities.

## 5. Conclusions

The results from this study indicated that the DCM extract of *A. australe* possesses antiplasmodial activity, which could be linked to the inhibition of the chaperone and ATPase functions of *Pf*Hsp70-1. The findings suggest that the DCM extract contains compounds that inhibit Hsp70 function, which could also target the Hsp70 functional cycle. The high antiplasmodial activity exhibited by the extract suggests the presence of highly potent antiplasmodial compounds within it. Using human cell lines, the future study will assess the cytotoxicity potential of the extract. This will be followed by in vivo testing of the antimalarial activity of the extracts using a malaria mouse model. Isolation of the responsible bioactive compounds as potential antimalarial drugs is also recommended for further study.

## Figures and Tables

**Table 1:** Major compounds found in the DCM extract of *A. australe*.

Peak Number	Retention Time (s)	Compound Name	Peak Area %	Molecular Formula	Molecular Weight (g/mol)	Bioactivities	Reference(s)
1	1496.4	n-Hexadecanoic acid	0.98394	C_16_H_32_O_2_	18	Larvicidal, anticancer, hemolytic	[20,21]
2	1635.8	Octadecanoic acid	0.91725	C_18_H_36_O_2_	25	Hepato-protective, antioxidant, larvicidal	[21,22]
3	1826.6	(S,Z)-Heptadeca-1,9-dien-4,6-diyn-3-ol	0.53102	C_17_H_24_O	244	Antitumor	[23]
4	1844.7	1,6-Heptadien-4-ol	1.9095	C_7_H_12_O	776	Antimicrobial	[24]
5	1990.8	1,6,10-Dodecatrien-3-ol, 3,7,11-trimethyl-	0.22794	C_15_H_26_O	698	Anti-parasitic, skin-repellent, antimalarial	[25,26,27,28]
6	2032.7	5-Benzofuranacetic acid, 6-ethenyl-2,4,5,6,7,7a-hexahydro-3,6-dimethyl-à-methylene-2-oxo-, methyl ester	22.564	C_16_H_20_O_4_	705	Anticonvulsant, anti-inflammatory, antimicrobial	[29]
7	2075.1	Methyl 10,12-pentacosadiynoate	2.3019	C_26_H_44_O_2_	729	Antimicrobial	[30]
8	2122.7	Fluoxymesterone	2.6697	C_20_H_29_FO_3_	561	Anticancer	[31]
9	2124.6	Estra-1,3,5(10)-trien-17á-ol	2.7174	C_18_H_24_O	645	Anti-estrogenic	[32]
10	2162.9	1-Eicosanol	0.57624	C_20_H_42_O	916	Antioxidant, fungicidal	[33,34]
11	2250.9	Stigmasterol	0.56558	C_29_H_48_O	869	Antibacterial, antifungal, anticancer	[35,36]

**Table 2:** Mortality (%) of the *P. falciparum* 3D7 cells following exposure to the plant extract.

Extract	Concentration (µg/mL)		IC_50_ (µg/mL)
	10	50	
*A. australe*	72.09 ± 3.84 **	94.84 ± 3.07 **	1.3 ± 0.11
Chloroquine	-	-	3.12

Chloroquine was used as a reference drug. Data are expressed as mean ± SD, (*n* = 3), ** *p* < 0.001 vs. normal control.

**Table 3:** Maximum absorbance of Hsp70-1 protein at 290 nm and 400 nm derived from UV-vis spectral data analysis of Figure 4.

Samples	Maximum Absorbance	
	290 nm	400 nm
*Pf*Hsp70-1 only (1 mM)	0.897	0.69
*Pf*Hsp70-1 with PMB (10 mM)	0.283	0.198
*Pf*Hsp70-1 with *A. australe* (5 µg/mL)	0.461	0.357
*Pf*Hsp70-1 with *A. australe* (10 µg/mL)	0.203	0.031

## Data Availability

The original contributions presented in this study are included in the article/Supplementary Materials. Further inquiries can be directed to the corresponding author.

## Supplementary Materials

The following supporting information can be downloaded at: https://www.mdpi.com/article/10.3390/microorganisms13092195/s1, Figure S1: (**A**) Expression of the *Pf*Hsp70-1 protein in *E. coli* BL21 cells: lane M-molecular weight marker (kDa); lane 0 HR-*Pf*Hsp70-1 expression without IPTG; lanes 1-6 HR and O/V-IPTG-induced *Pf*Hsp70-1 expression. (**B**) Western blot of the expressed *Pf*Hsp70-1. (**C**) Purification of the *Pf*Hsp70-1 protein; lane CL, *Pf*Hsp70-1 clear lysate, lane W1-W4 wash samples, and lane E1–E4 were the eluted *Pf*Hsp70-1 protein. (SEE Figure 1, main Article). Figure S2: Antiplasmodial activity of the *australe* DCM extract. The antiplasmodial activity of the extract was determined against *P. falciparum* strain 3D7. Percentage viability of the parasite was plotted against the extract concentration (Log) and the IC_50_ (50% inhibitory concentration) was obtained from the resulting dose-response curve by non-linear regression. Concentration used: 100 μg/mL down in 3-fold dilutions (100 μg/mL–0.0457 μg/mL). Chloroquine was used as a reference drug. Data are expressed as mean ± SD, (*n* = 3).

## Abbreviations

DCM	Dichloromethane
GAE	Gallic Acid Equivalent
GC-MS	Gas chromatography–mass spectrometer
IPTG	Isopropyl-β-D-thiogalactopyranoside
MDH	Malate dehydrogenase
PfHsp	Plasmodium falciparum heat shock protein
PMB	Polymyxin B
QE	Quercetin
SDS-PAGE	Sodium dodecyl sulfate polyacrylamide gel electrophoresis
UV-vis	Ultraviolet-visible (UV-vis)
