# Supplementary material for: Acanthospermum australe Extract Inhibits the Chaperone Activity of Plasmodium falciparum Heat Shock Protein 70-1
Source: Microorganisms. 2025 Sep 19;13(9):2195. doi: 10.3390/microorganisms13092195 (PMC12472254; doi:10.3390/microorganisms13092195)
Supplement: Supplementary file 1 [file microorganisms-13-02195-s001.zip › microorganisms-3750792-supplementary.pdf]

Acanthospermum australe Extract Inhibits the Chaperone Activity of *Plasmodium falciparum* Heat Shock Protein70-1

Ntombikhona Appear Koza* Ntokozo Nkosinathi Myeza, Heinrich Hoppe, Rebamang Anthony Mosa, Abidemi Paul Kappo, Mthokozisi Blessing Cedric Simelane and Andrew Rowland Opoku

1. Uncropped and unadjusted SDS-PAGE gels and Western blot gels

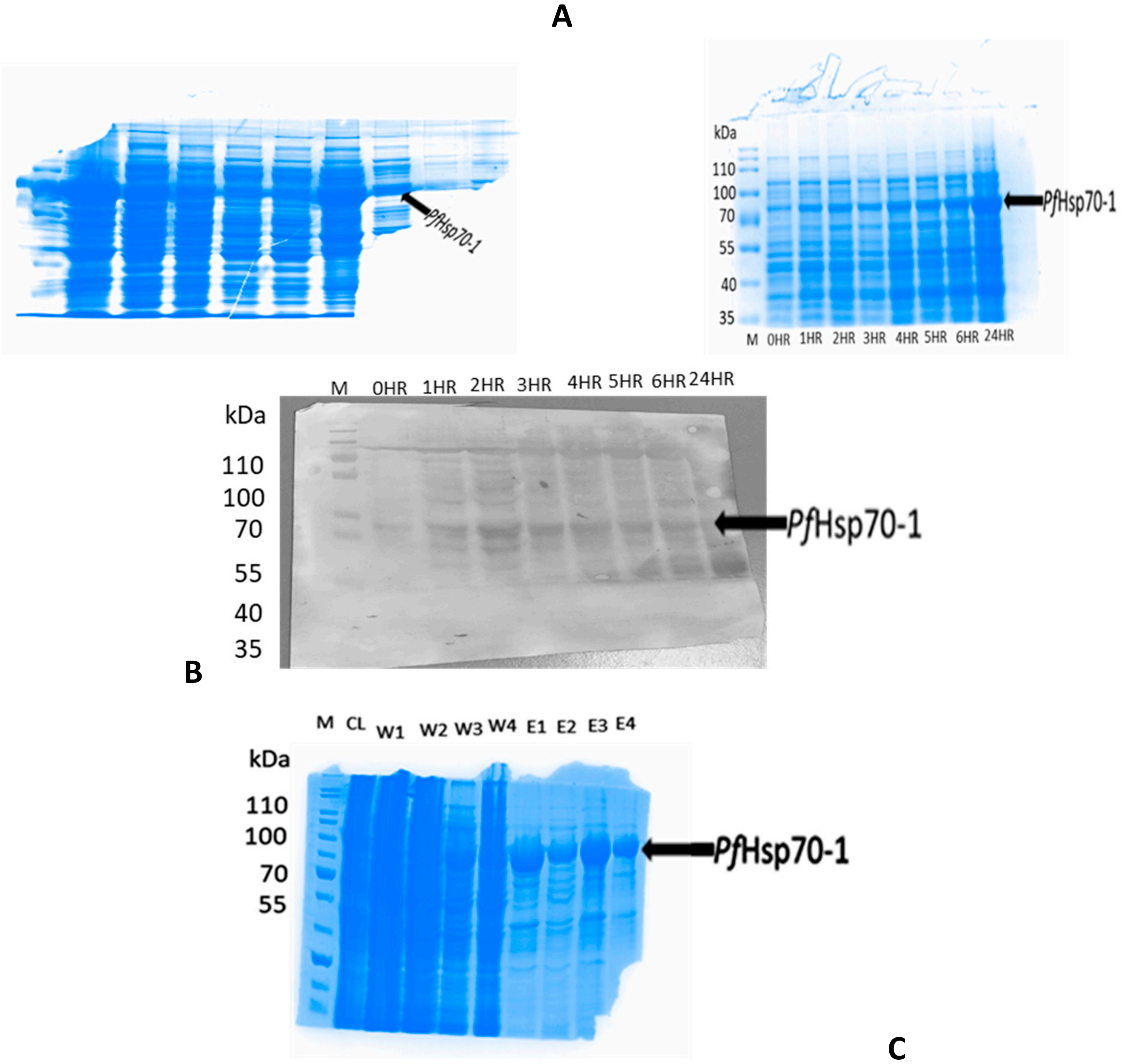

Figure S1. (**A**) Expression of the *Pf*Hsp70-1 protein in *E. coli* BL21 cells: lane M-molecular weight marker (kDa); lane 0 HR-*Pf*Hsp70-1 expression without IPTG; lanes 1-6 HR and O/V-IPTG-induced *Pf*Hsp70-1 expression. (**B**) Western blot of the expressed *Pf*Hsp70-1. (**C**) Purification of the *Pf*Hsp70-1 protein; lane CL, *Pf*Hsp70-1 clear lysate, lane W1-W4 wash samples, and lane E1–E4 were the eluted *Pf*Hsp70-1 protein. (SEE Figure 1, main Article)

2. Dose-response curve (non-linear) used to determine the IC_{50} of the *A. australe* extract

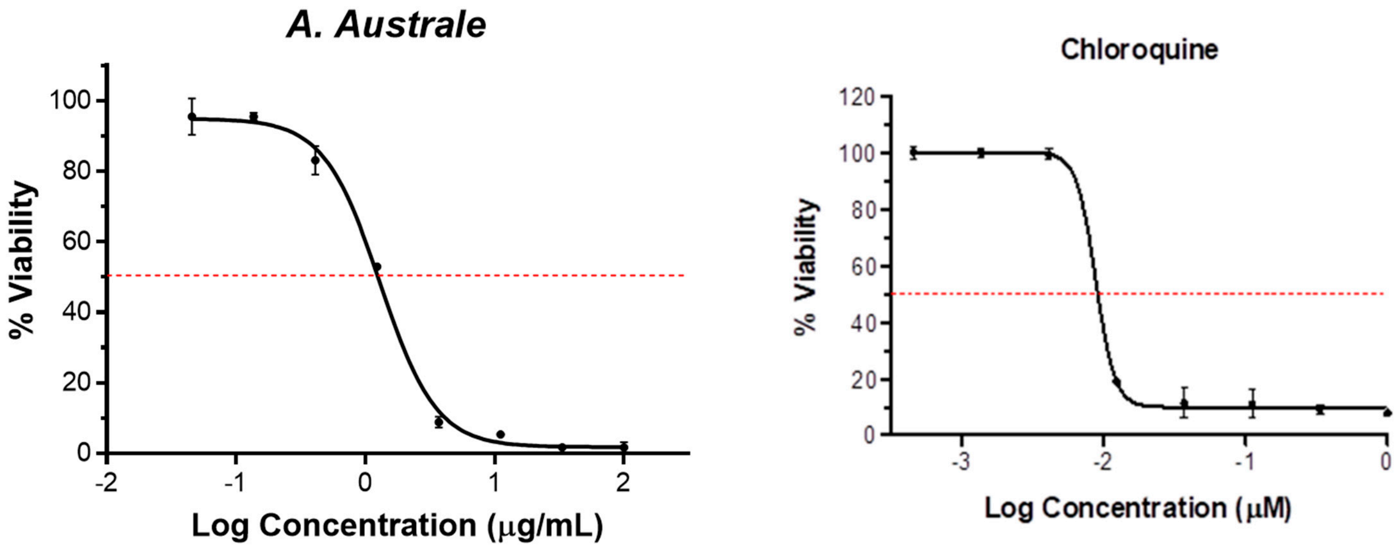

		A. Australe	
Conc (µg/mL)	Log (Conc)	% Viab	SD
100	2	1.73	1.44
33.33	1.523	1.73	1.00
11.11	1.046	5.34	0.83
3.704	0.568	8.86	1.53
1.235	0.091	52.90	0.89
0.412	-0.385	83.07	4.03
0.137	-0.863	95.47	1.12
0.0457	-1.34	95.47	5.14

Figure S2. Antiplasmodial activity of the *australe* DCM extract. The antiplasmodial activity of the extract was determined against *P. falciparum* strain 3D7. Percentage viability of the parasite was plotted against the extract concentration (Log) and the IC_{50} (50% inhibitory concentration) was obtained from the resulting dose-response curve by non-linear regression. Concentration used: 100 µg/mL down in 3-fold dilutions (100 µg/mL – 0.0457 µg/mL). Chloroquine was used as a reference drug. Data are expressed as mean ± SD, (*n* = 3).
